# Deep learning for COVID-19 detection based on CT images

**DOI:** 10.1038/s41598-021-93832-2

**Published:** 2021-07-12

**Authors:** Wentao Zhao, Wei Jiang, Xinguo Qiu

**Affiliations:** 1 College of Mechanical Engineering, Zhejiang University of Technology, Hangzhou, 310023 China; 2 School of Intelligent Transportation, Zhejiang Institute of Mechanical & Electrical Engineering, Hangzhou, 310053 China

**Keywords:** Mathematics and computing, Engineering

## Abstract

COVID-19 has tremendously impacted patients and medical systems globally. Computed tomography images can effectively complement the reverse transcription-polymerase chain reaction testing. This study adopted a convolutional neural network for COVID-19 testing. We examined the performance of different pre-trained models on CT testing and identified that larger, out-of-field datasets boost the testing power of the models. This suggests that a priori knowledge of the models from out-of-field training is also applicable to CT images. The proposed transfer learning approach proves to be more successful than the current approaches described in literature. We believe that our approach has achieved the state-of-the-art performance in identification thus far. Based on experiments with randomly sampled training datasets, the results reveal a satisfactory performance by our model. We investigated the relevant visual characteristics of the CT images used by the model; these may assist clinical doctors in manual screening.

## Introduction

The year 2019 witnessed the outbreak of a viral pneumonia, originating from an unknown source in Wuhan, China. The virus was soon termed severe acute respiratory syndrome coronavirus 2 (SARS-CoV-2) by the World Health Organization^1–3^, and the resulting pneumonia is called Coronavirus disease 2019 (COVID-19)^2–6^. To date, more than 120 million cases have been confirmed worldwide, and the number is still on the rise. Currently, the reverse transcription-polymerase chain reaction (RT-PCR) which relies on nasopharyngeal swabs to examine the existence of the ribonucleic acid (RNA) of SARS-CoV-2^7^ is still a popular approach to test for the disease. Despite the high level of specificity (Sp) of testing with RT-PCR, the sensitivity (Sn) of the method could be relatively low^8,9^, and there is significant variability in efficacy depending on different sampling methods and the time of occurrence of symptoms^9,10^. Apart from the confirmation by pathogenic labs, other useful methods for COVID-19 diagnosis include examination of clinical characteristics and the use of computed tomography (CT) imaging^11,12^. Owing to its high sensitivity, CT imaging has been proposed as an essential substitute tool for COVID-19 screening, which is especially effective as a complementary method to RT-PCR, and by way of CT imaging was able to perform rapid prediction compared to RT-PCR. In particular, researches has shown that in normal CT scans conducted for non-COVID-19 reasons, such as for examination before elective operations and nerve system examinations, CT is considerably useful in testing for COVID-19 infection^13,14^. In other cases where CT imaging is adopted, for example, when patients suffer from worsening respiratory complications or similar factors, and are tested negative using RT-PCR, the clinical evidence may show patterns similar to being positive for COVID-19. Early research has suggested that CT images contain a great number of potential indicators of infection^8,10^, but that the infection could also be unrelated to COVID-19. This implies some challenges for radiologists in specifically identifying COVID-19 infections using CT images^15,16^. In addition, visual analysis of CT images is also time-consuming, especially in large-scale studies or with a huge number of patients. A common problem in the analysis of medical images has been that most of the CT images used for diagnostic purposes are not openly accessible owing to privacy concerns, which means that the results from neural network training on any particular one dataset cannot be replicated or applied in other hospitals. The absence of open-source datasets on COVID-19 CT images thus presents a tremendous obstacle for the development of more advanced artificial intelligence technologies for better detection of CT on COVID-19 testing^17^. With the urgent need for solutions to cope with the COVID-19 pandemic and based on the recent efforts among researchers to promote open-source data and open access^18,19^, we discussed how transfer learning can improve the performance of convolutional neural networks on COVID-19 testing using CT images, and found that pre-trained models trained on larger out-of-domain datasets have better performance in COVID-19 detection. Comparing the model architecture which was discovered automatically via a machine-driven design exploration process using generative synthesis, our model performs better in each evaluation metric. We aimed to make the following contributions:

- We used various training steps, resolutions with and without mixup to test the impact of these hyperparameters on the results and discovered that a higher resolution and an appropriate number of training steps are effective in raising the model performance. As the model itself already yields excellent results, provided the data are sufficient, there is little impact of implementing mixup on the results.
- With five different strategies for parameter initialization in the models, we studied the impact of initialized parameters on the model performance. Our results demonstrate that different pre-training parameters influence the final performance of fine-tuned models. By utilizing a larger out-of-field dataset for pre-training, the model can be more effectively generalized.
- By comparing our results with those from previous studies, we demonstrate that our models based on transfer learning are better than those based on structural design and that our models achieve state-of-the-art performance. Furthermore, we evaluated the performance of our model in a case in which there was a small quantity of downstream data and found that it still showed excellent performance in identifying COVID-19.
- With visualization, we investigated the mechanism behind the model for COVID-19 testing to better aid clinical decision-making.

## Related work

### COVID-19 research

Currently, research on COVID-19 is being effectively carried out in various areas. Reference^20^ review the various types of scalable telehealth services used to support patients infected by COVID-19 and other diseases. Reference^21^ discuss the different wearable monitoring devices and respiratory support systems which are frequently used to assist coronavirus affected people. Reference^22^ present an overview of the existing technologies, which are frequently used to support the infected patients for respiration. They outline a comparative analysis among the developed devices necessary challenges and possible future directions for the proper selection of affordable technologies. Reference^23^ propose a system that restricts the spread of COVID-19 by detecting people not wearing any facial mask in a smart city network.

In the face of the potential for using CT images as a complementary screening method for COVID-19, alongside the challenges of interpreting CT for COVID-19 screening, extensive studies have been conducted on how to detect COVID-19 using CT images. Deep learning is now widely used in all aspects of COVID-19 research aimed at controlling the ongoing outbreak^24–28^, reference^29^ give an overview of the recently developed systems based on deep learning techniques using different medical imaging modalities such as CT and X-ray. Reference^17^ established a database of hundreds of CT scans of COVID-19 positive cases and developed a deep learning approach with high sample efficiency based on self-supervision^30^ and transfer learning^31^. In addition, researchers have developed an artificial intelligence system capable of diagnosing COVID-19 and separating the disease from the other common pneumonia as well as the normal cases^32^. Furthermore, reference^33^ created a library containing CT images of 1,521 pneumonia patients (including those with COVID-19), 130 clinical symptoms (a series of symptoms including biochemical and cellular analysis of blood and urine), as well as the clinical symptoms of SARS-CoV-2, and made predictions on whether each patient experienced negative, mild, and severe cases. With machine-driven design exploration, reference^34^ proposed a deep convolutional neural network structure, COVIDNet-CT, based on CT images. Similarly, leveraging 104,009 CT images from 1,489 patients collected from the China National Center for Bioinformation (CNCB) (China)^32^ combined with data cleaning and preparing in a suitable format for benchmarking, a COVIDx-CT dataset was built, along with explainability-driven performance validation and analysis using the GSInquire technology^35^. Building upon the above progress, researchers proposed the COVIDx CT-2 datasets, which increases the number and diversity of patients^36^.

### Transfer learning

Transfer learning is the cornerstone of computer vision. Various categorization tasks related to images^37^ can achieve greater performance with datasets of a limited size with transfer learning than using any other method. Previous work has shown that effective performance can be achieved through pre-trained models fine-tuned on specific tasks^38,39^.

## Methods

### Datasets

With the global spread of the COVID-19 pandemic, accessibility of first-hand CT images and clinical data is critical for guiding clinical decisions, providing information which can deepen our understanding of the patterns of infection by the virus, and offering systematic models for early diagnosis and timely medical interventions. A key approach is to establish a comprehensive database with open access to CT images and associated clinical symptoms to facilitate the global fight against COVID-19. As mentioned in Related work section, several datasets have been built and are open for researchers, doctors, and data scientists for COVID-19-related research. Currently, although the COVIDx-CT dataset is evidently larger than many other CT datasets used in the literature on COVID-19 testing, a potential limitation of using COVIDx-CT for deep neural network learning lies in the limited patient demographic diversity. Specifically, as COVIDx-CT is collected from the CNCB, only information from the different provinces in China is available, meaning the symptoms of COVID-19 in the CT images may not be appropriately generalizable to cases beyond China. Increasing the number and diversity of patients would make deep neural networks more varied and comprehensive, so that they can be more generalizable and applicable in different clinical environments around the world. By carefully processing and organizing the CT images of patients based on various CT devices, solutions, and validation abilities, previous researchers^36^ established the COVIDx CT-2A and COVIDx CT-2B datasets. COVIDx CT-2A involves 194,922 images from 3,745 patients aged between 0 and 93, with a median age of 51. Each CT scan per patient has many CT slides. We use the CT slides as the input images to detect COVID-19, making the COVID-19 detection problem an image classification problem. The CT images are provided as $512 \times 512$ pixels. The sources of input for the images in COVDx CT-2A are as follows:

- China National Center for Bioinformation (CNCB) (China)^32^
- National Institutes of Health Intramural Targeted Anti-COVID-19 (ITAC) Program (countries unknown)^40^
- Negin Radiology Medical Center (Iran)^41^
- Liyuan Hospital and Wuhan Union Hospital of Tongji Medical College of Huazhong University of Science and Technology (China)^33^
- COVID-19 CT lung and infection segmentation project, annotated and verified by Nanjing Drum Tower Hospital (China)^42^
- Lung Image Database Consortium (LIDC) and Image Database Resource Initiative (IDRI) (countries unknown)^43^
- Open access online collaborative radiology resource (Radiopaedia) (countries unknown)^44^

**Figure 1:**
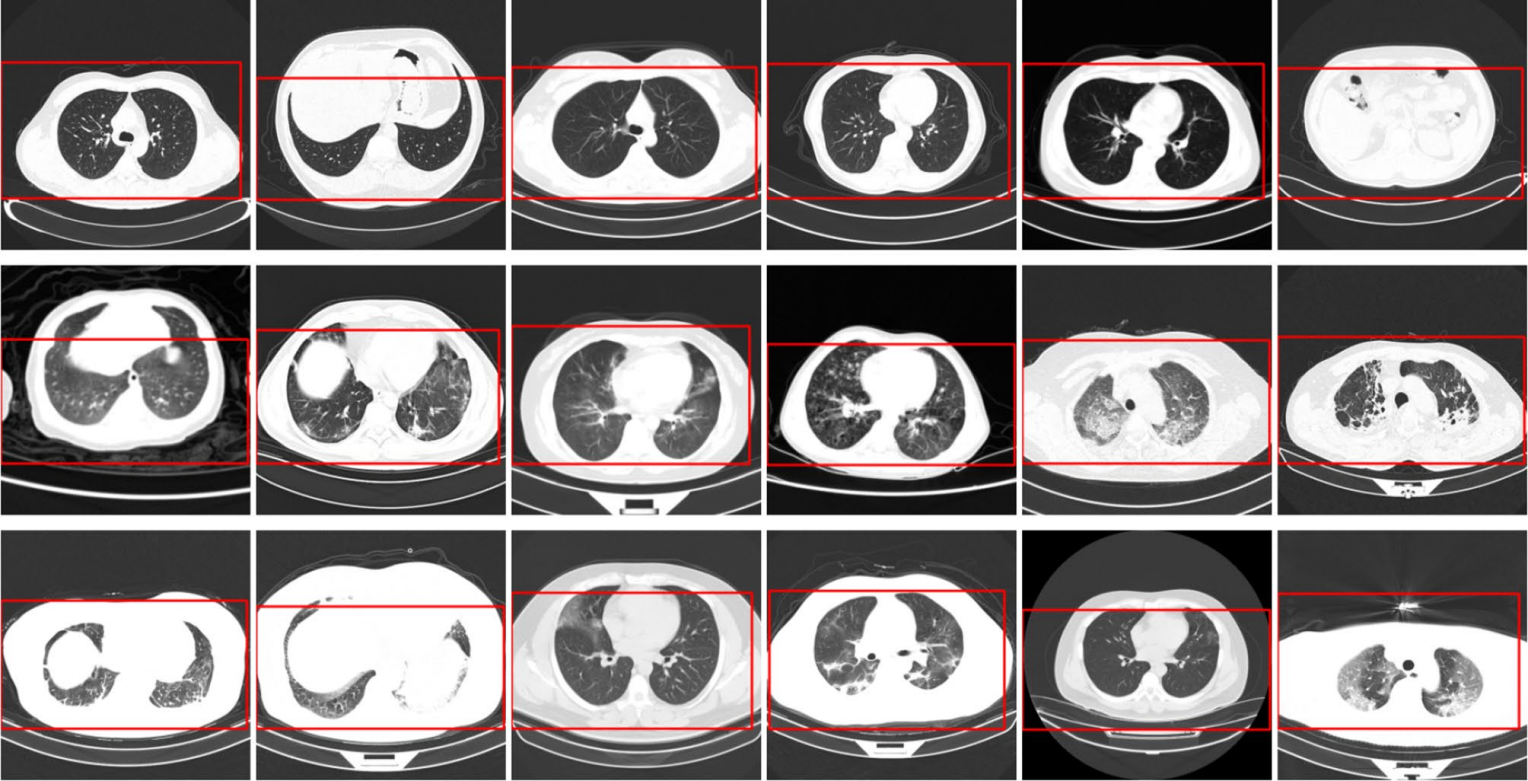
Relevant examples of CT images in COVIDx CT-2. The red frames refer to the marker frames of CT images in the dataset. The first line shows the normal controls, the second line shows the cases with CP, and the third line shows the cases with NCP infected by SARS-CoV-2.

**Figure 2:**
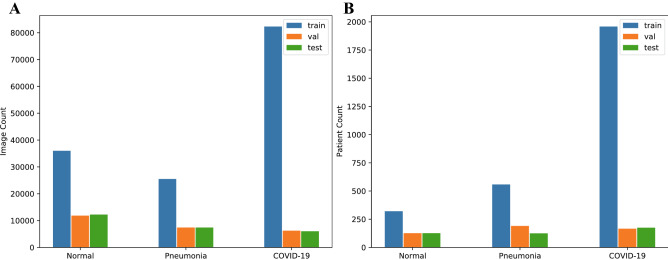
COVIDx CT-2A dataset training, testing and validation dataset. (**A**) CT image distribution. (**B**) patient distribution.

Building upon COVIDx CT-2A, COVIDx CT-2B augmented the dataset with weak validation (MosMed) from the Research and Practical Clinical Center of Diagnostics and Telemedicine Technologies, Department of Health Care of Moscow (Russian Federation)^45^. The purpose of establishing this validation set is to investigate, for instance, whether adding weak validation (i.e., findings without using RT-PCR and radiological tests) training data would boost the performance of the model. This validation can further increase the breadth and diversity of the dataset. In view of the comparison with previous working models and the openness of data, in the present study we employed COVIDx CT-2A for COVID-19 testing. Figure 1 illustrates the relevant examples in the COVIDx CT-2A dataset, including 3 types of CT scans: novel coronavirus pneumonia (NCP) infected by SARS-CoV-2, common pneumonia (CP), and normal controls. We applied some modifications to images from the database to facilitate our models. Specifically, as the potential contrast in the background of the images may result in biases in the models, we removed the background with an automatic cropping algorithm to standardize the field to the body area (as shown by the red frames in Fig. 1). By means of comparison across various types, we identified the ground glass opacity (GGO), lung consolidation^46^, and even the presence of white pneumonia in the groups of CP and NCP. However, owing to the considerably subtle visual differences in the images between those infected with common pneumonia and those infected with SARS-CoV-2, there might be tremendous variations in the ability to distinguish between the diseases, even for radiologists. Figure 2 presents the distribution of the different types of infections and images in training, test, and validation sets.

### Model selection

With the design exploration mode forming with machine-driven generation, previous researchers^34^ have designed the deep convolutional neural network COVID-Net-CT for COVID-19 testing based on CT images. The subsequent COVID-Net CT-2^36^ was then designed using this architecture as its basis. In our experiment, we adopted the ResNet-v2, which is a modified version from ResNet^47^. Next, we substituted group normalization^48^ for batch normalization^49^ and conducted a weight standardization^50^ for all convolutional layers. To investigate how transfer learning utilizes external data in COVID-19 testing based on CT images, we incorporated the pre-training data from CIFAR-10^51^, ILSVRC-2012^52^, and ImageNet-21k^53^ as the parameters for initialization to train the models.

### Hyperparameter settings for training

**Figure 3:**
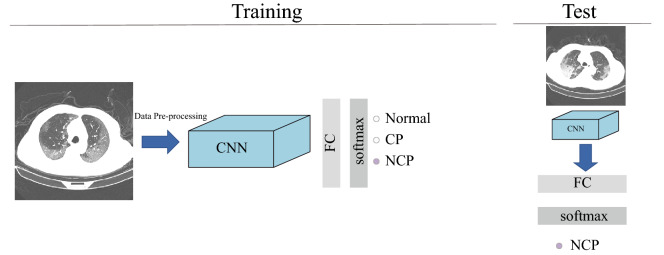
A general flowchart of deep learning based COVID-19 diagnosis system.

The general flowchart of the COVID-19 diagnosis system based on deep learning is illustrated in Fig. 3. The total system contains two sections. In the training section, the training data are used to update the model parameters, and the performance of developed model is appraised by test data. In the test section, the model can be used to extract the feature, and finally identify the class labels based on the feature. Lastly, the developed model is assessed by some evaluation metrics like accuracy, sensitivity, specificity, and so on.

The pseudocode for fine-tuning the Convolutional Neural Network (CNN) and obtaining the accuracy can be seen in Algorithm 1. For each iteration, we randomly selected *b* CT images to calculate the gradient and updated the network parameters. Unlike the previous standard training process, we did not constrain the epoch of iteration, but constrained the training steps instead. Regarding the choice of hyperparameters, we used the stochastic gradient descent (SGD) and set the learning rate at 0.003, the momentum at 0.9, and the batch size at 64. RGB reordering was applied, and the final input to the proposed model was provided as $512 \times 512 \times 3$ image. Concerning data augmentation, for the training set we first tailored the images according to the annotated cropping frame, and then adjusted them to $512 \times 512$ pixels, randomly segmented them to $480 \times 480$ pixels, followed by random horizontal flips and normalization. For the test set, we simply adjusted the images that were cropped according to the annotation, and then resized them to $480 \times 480$. We used 10,000 training steps in our experiments. To fine-tune the model, we first conducted a warmup^54^ for the learning rate, and then reduced the learning rate three times at a rate of 10x during the entire training. The details are provided in Parameter sensitivity section. Finally, we used mixup (Eq. (1)) for data augmentation.

1 $$\begin{aligned} \left\{ \begin{array}{ll}{\tilde{x}}=\lambda x_{i}+(1-\lambda ) x_{j}, \\ {\tilde{y}}=\lambda y_{i}+(1-\lambda ) y_{j}, \end{array}\right. \end{aligned}$$

Here, $x_{i}$ and $x_{j}$ are the initial input vectors, while $y_{i}$ and $y_{j}$ are the labels. Through mixup, we obtained new vectors and labels. As the calculation of loss using cross entropy is a convex optimization problem, the convex optimization problem has good convergence properties when solved by gradient descent, we used cross entropy as the loss function (Eq. 2).

2 $$\begin{aligned} \begin{aligned} {\text {loss}}(x, \text{ class } )&=-\log \left( \frac{\exp (x[\text {class}])}{\sum _{j} \exp (x[j])}\right) \\&=-x[\text {class}]+\log \left( \sum _{j} \exp (x[j])\right) \end{aligned} \end{aligned}$$

where $x \in {\mathbb {R}}^{N \times C}$ is the output of the model, $\text{ class } \in {\mathbb {R}}^{N}$ is the label of the CT imaging and $0 \le {\text {class}}[i] \le C-1$.

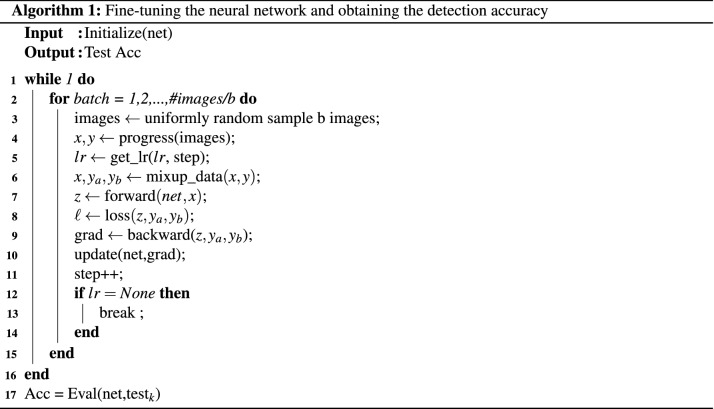



## Results

In this section, we investigate the model performance in testing for COVID-19. Specifically, we endeavor to address the following questions:

- How are different hyperparameters, including various resolutions, training steps, and mixup, used to affect the model performance?
- How do different weight initializations trained from the different datasets affect result?
- Can we obtain a satisfactory result with the proposed model with limited CT images?
- How can we understand the decisions made by the deep convolutional neural network to assist in clinical decision-making?

### Test performance

**Figure 4:**
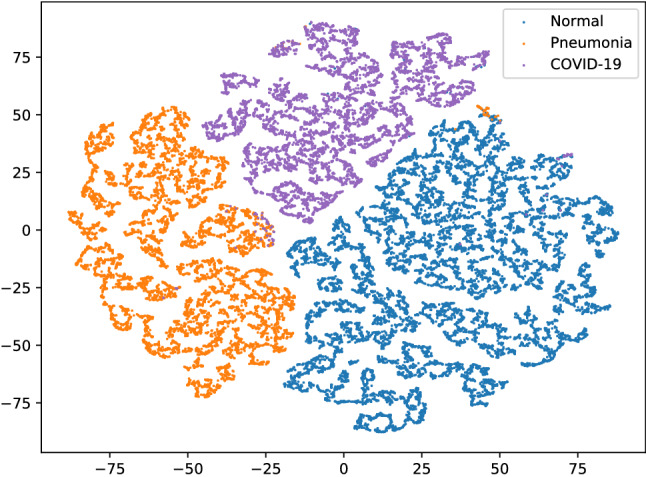
Distribution of characteristics of CT images after dimensionality reduction with t-SNE^57^. Each node refers to a different CT image, the color reflects the information on categories, and the meaning of the color is defined in the legend.

**Figure 5:**
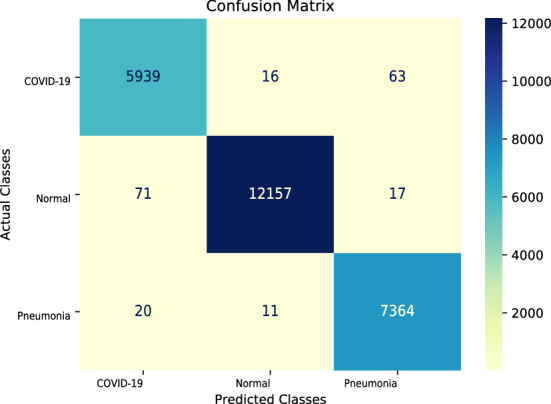
Confusion matrix for COVID-19 testing using Bit-M fine-tuned model. The color bar indicates the intensity of normalization.

We utilized the training setting described in Hyperparameter settings for training section to train the models. The results are summarized in Table 1 and are compared with those from the current most advanced methods. Random, Bit-S and Bit-M are the models adopted in our laboratory, and refer to the random initialization, and methods of pre-training on ILSVRC-2012 and ImageNet-21k, which will be introduced in Impact of parameter initialization section. We compared our model with the most advanced COVID-Net CT-2 L. Table 1 reveals that our Bit-S and Bit-M models which rely on transfer learning saw an increase in accuracy of 0.71% and 1.12% over COVID-Net CT-2 L model, respectively. In addition, the accuracy of our model of random initialization was 3.60% higher than that of COVID-Net CT-1, suggesting that in comparison with models using structure space search, our model with random parameter initialization also has excellent performance. Figure 4 shows the distribution of the CT images representations after dimensionality reduction, which highlights the proper differentiation of the different categories. In the confusion matrix^55^ in Fig. 5, we demonstrate that even though radiologists may sometimes fail to distinguish between CP and NCP, our model provides accurate classifications. For a better quantitative analysis of the models, four indicators were introduced, namely sensitivity (Sn), specificity (Sp), positive predictive value (PPV), and negative predictive value (NPV), as summarized in Table 2. We discovered that the BiT-M model based on transfer learning achieved the state-of-the-art performance with respect to sensitivity for COVID-19 (98.7%), positive predictive value (98.5%), specificity (99.5%), and negative predictive value (99.6%). Our proposed technique outperforms previous works because we pre-train the model on a larger out-of-domain dataset which enables the model to learn more generalize knowledge. From a clinical perspective, high sensitivity ensures that there are few false negatives that lead to missed diagnoses in patients with COVID-19 infection, and high PPV ensures few false positives which add an unnecessary burden on the health care system. High specificity and NPV achieved by our Bit-M model ensure that COVID-19 negative predictions are indeed true negatives in the vast majority of cases, the prediction results are real and reliable for COVID-19 negative patients. The problem of treating false positives and false negatives is equivalent, specifically, we cannot afford to diagnose a COVID-19 positive patient as negative, as in this case, the patient may go back into community, believing to be free of COVID-19, which leads to community transmission of the disease^56^. When we diagnose too many COVID-19 negative as positive, it increases the burden on the healthcare system and causes public panic. Psychological stress my result if a negative person is diagnosed as positive.

**Table 1:** Accuracy of the COVIDx CT-2A benchmark datasets.

Model	Methods	Accuracy(%)
COVID-Net CT-1	Structure design	94.5
COVID-Net CT-2 L	Structure design	98.1
COVID-Net CT-2 S	Structure design	97.9
Random (ours)	Transfer learning	97.9
Bit-S (ours)	Transfer learning	98.8
Bit-M (ours)	Transfer learning	**99.2**

**Table 2:** Sensitivity, PPV, Specificity, and NVP of the test data in COVIDx CT-2A benchmark datasets.

Network	Sensitivity (%)			PPV (%)			Specificity (%)			NPV (%)		
	Normal	CP	NCP	Normal	CP	NCP	Normal	CP	NCP	Normal	CP	NCP
COVID-Net CT-1	98.8	99.0	80.2	96.1	90.2	97.6	96.3	95.7	99.4	98.9	99.6	94.2
COVID-Net CT-2 L	99.0	98.2	96.2	99.4	97.2	96.7	99.5	98.8	99.0	99.1	99.3	98.8
COVID-Net CT-2 S	98.9	98.1	95.7	99.3	97.0	96.4	99.3	98.8	98.9	99.0	99.2	98.7
Random(ours)	97.9	98.6	96.9	98.9	97.5	96.4	99.0	99.0	98.9	98.1	99.4	99.1
Bit-S(ours)	99.0	99.3	97.9	99.6	97.9	98.4	99.6	99.1	99.5	99.1	99.7	99.4
Bit-M(ours)	99.3	99.6	98.7	99.8	98.9	98.5	99.8	99.6	99.5	99.3	99.8	99.6

### Hyperparameters sensitivity

**Figure 6:**
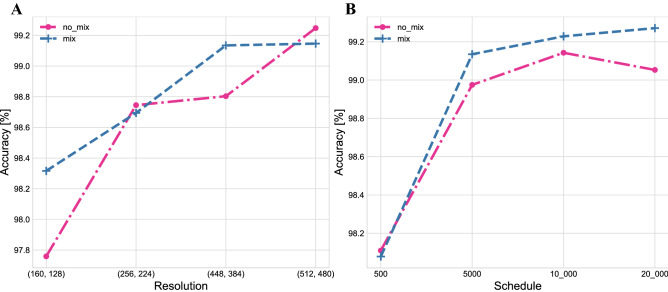
Test accuracy of COVIDx CT-2 with various hyperparameters. (**A**) Resolution. (**B**) Schedule.

In this section, we explore the effect of various hyperparameters on model performance, specifically: training steps, image resolutions, and whether to use mixup. We used four combinations of overall training steps and input resolutions. For the resolutions of CT images, we adopted the settings (160, 128), (256, 224), (448, 384), and (512, 480), where the first value in each doublet indicates the scale of adjustment during training, while the second value indicates the size of random cropping during training and testing. Regarding the length of the training project, we used [100, 200, 300, 400, 500], [500, 1,500, 3,000, 4,500, 5,000], [500, 3,000, 6,000, 9,000, 10,000], and [500, 6,000, 12,000, 18,000, 20,000]. The first parameter refers to the number of steps in the warmup step, the last parameter is the end step, and the rest are the step nodes with a learning rate decaying by 10 times. Figure 6 displays the test accuracy for different resolutions and training steps with and without mixup. The results emphasize that a higher resolution can increase accuracy in identification, which means that clearer CT images contain more diagnostic clinical information. A larger training step can also improve accuracy, but the effect is less significant when it exceeds 10,000. The results suggest that for resolutions of (512, 480) and a training step of 10,000 between Fig. 6a and  b, the accuracy rates are exactly the opposite (The hyperparameter settings for the experiments are the same). This phenomenon is a result of the random sampling. It indicates that the performance of the model is not enhanced by the mixup due to the data being already rich enough.

### Impact of parameter initialization

**Figure 7:**
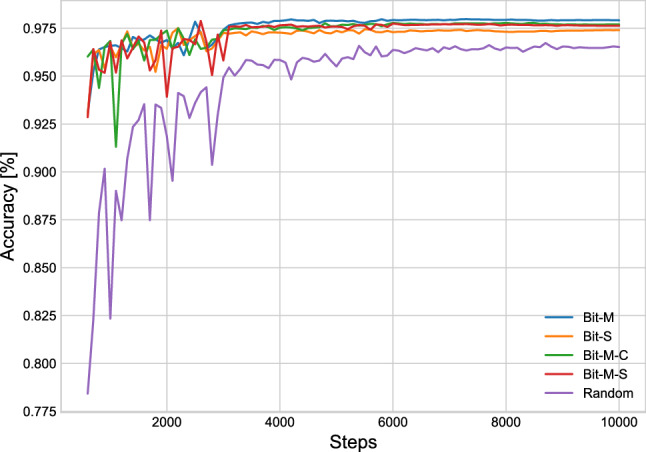
Validation accuracy curves of various initialized models.

To evaluate the impact of parameter initialization on the task performance, we used the pre-trained ResNet50x1 models to investigate how upstream pre-training can affect the fine-tuning performance. Random means the parameters were randomly initialized in the models. BIT-M was pre-trained on the complete ImageNet-21k dataset, a public dataset with 14,200,000 images and 21,000 categories. The images could contain multiple labels. BIT-S was pre-trained on the ILSVRC-2012 variant from ImageNet, which include 1,280,000 images and 1,000 categories. BIT-M-S was first pre-trained on the ImageNet-21k dataset and then fine-tuned on ILSVRC-2012. BIT-M-C first went through pre-training using the ImageNet-21k dataset and was then fine-tuned on CIFAR-10 which contains 60,000 images ($32 \times 32$ pixels) across 10 categories. The weight initialization was pre-trained on out-of-domain data from a previous study^58^. For a fair comparison, we set the training step as 10,000 and used mixup, while the other settings were the same as those in Hyperparameter settings for training section. The impact of weighting initialization is illustrated in Table 3. We repeated the experiment and the results were slightly different from Table 1 because of random sampling and random initialization of model parameters. We realized that the parameter pre-trained on ImageNet-21k exhibited better performance in generalization compared to that pre-trained on ILSVRC-2012. Meanwhile, this performance would not be affected even by the fine-tuning on out-of-field datasets. Afterwards, we calculated the test performance for every 100 steps, presented in Fig. 7. The models pre-trained on ImageNet-21k (BIT-M, BIT-M-S, and BIT-M-C) exhibited better performance in the evaluation with the test set at later stages than did the ILSVRC-2012 initialized weighting (BIT-S). This result highlights that training with the larger dataset results in greater generalizability.

**Table 3:** Categorization accuracy of test and validation sets with different weight initialization.

Weight initialization	Val	Test
Random	96.5	97.7
Bit-S	97.4	98.8
Bit-M	97.9	99.3
Bit-M-C	97.7	99.2
Bit-M-S	97.6	98.9

### Influence of the size of labeled training data on model performance

**Figure 8:**
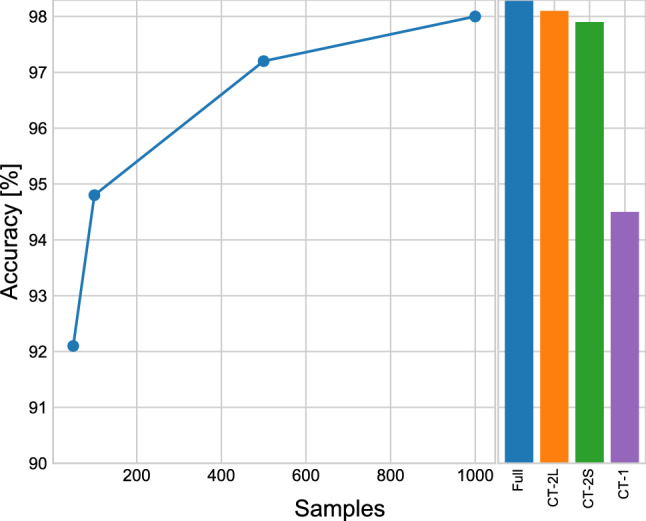
Impact of the number of each category of images in the training set on the performance of the model on the test set.

To evaluate how the models perform on the small downstream datasets akin to those which would be used in real-world situations, a certain number of images from each category were randomly selected for a performance test. For each category, we randomly chose 50, 100, 500, and 1,000 samples for training and tested the trained model to see the identification rate with the test set. The results of these tests were presented in Fig. 8. The histogram on the right showed the outcomes of the Imagenet21k pre-trained model using the entire training set, CT-2L, CT-2S, and CT-1. When conducting these tests, we noticed that BIT-M achieved a higher test accuracy with a limited number of labeled images. When 100 images were selected from each category, the accuracy (94.8%) already exceeded that of the experimental result using CT-1 (94.5%). When 1,000 images were selected, the accuracy (98.0%) was as good as that of CT-2S (97.9%). This lends support to the immense potential of our transfer learning models, which can still function well using limited dataset. This suggests that the priori knowledge learned through pre-training on large, out-of-field datasets can still ensure an excellent performance in the case of limited training data.

### Qualitative analysis of Covid-19 testing of the model

**Figure 9:**
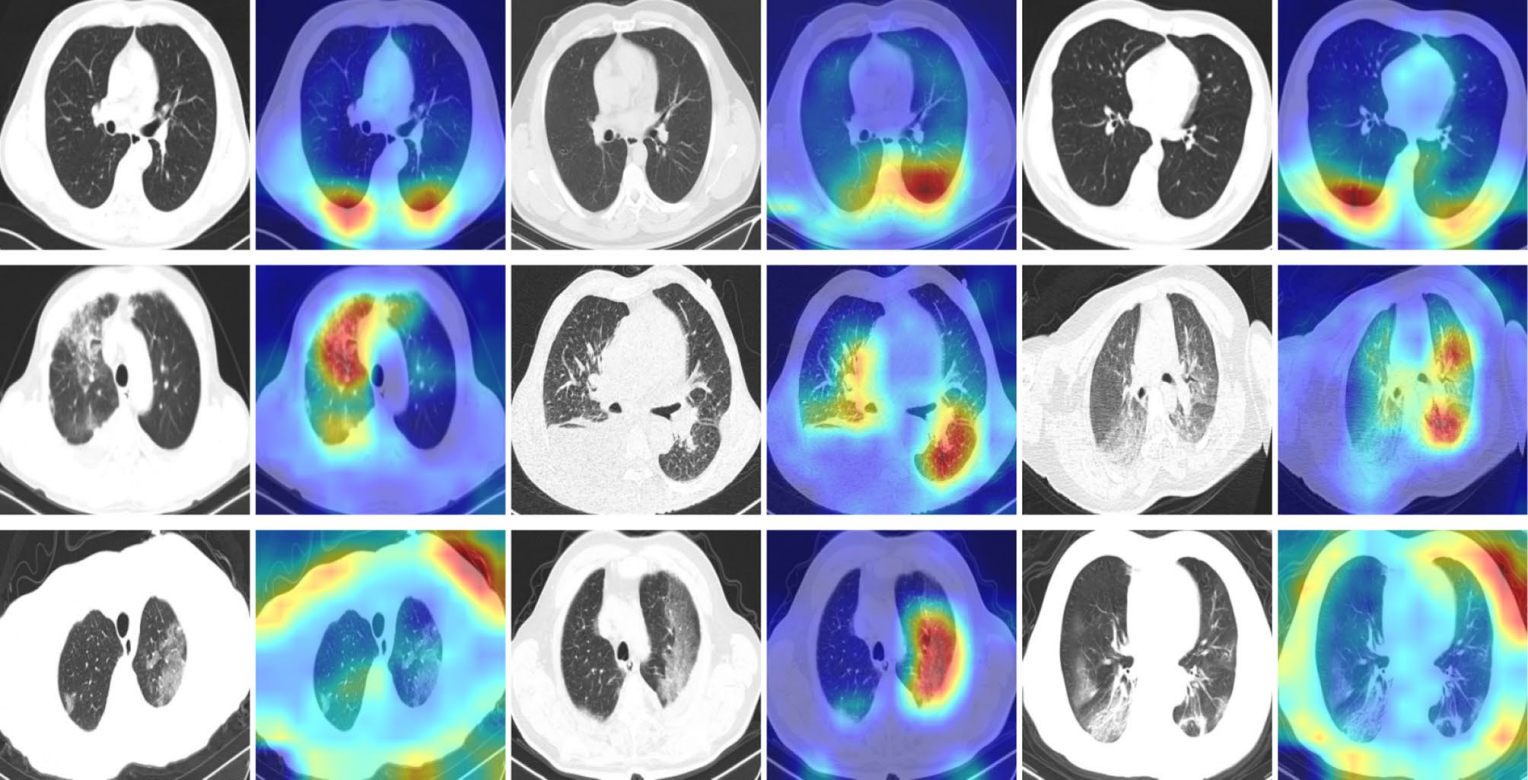
Grad-CAM visualization of Bit-M. The first line depicts the normal case, the second line the case of CP, and the third line the case of COVID-19 (NCP) due to infection by SARS-CoV-2. The model using CT images as input source have yielded accurate predictions.

Although performance indicators are useful for model evaluation, they fail to explain the decision-making behavior of the network. In this regard, we employed the Grad-CAM^59^ visualization technique to explore the areas of concern for the models in COVID-19 testing, to better understand which characteristics of CT images are key for diagnostic accuracy, and thus aid clinical decision-making. As demonstrated in Fig. 9, we first cropped the images using the detection frame (introduced in Hyperparameter settings for training Section), enlarged them to $480 \times 480$ pixels, and used Grad-CAM for visual explanation. All the predictions of the model using CT images in Fig. 9 are the same as the actual detection results. In most cases, the performance of the model is the same as would be expected for typical human visual cognition. This is particularly true for CP, as the model successfully focus on the disease areas, and display the affected regions of lungs. The radiologist further can apply color visualization approach using Grad-CAM for making efficient and confident decision^60^. For the norm case, the model focuses more on the lower region. Although NCP due to SARS-CoV-2 could be detected using the first and third CT images (third row in Fig. 9), the model was more interested in the texture at the periphery. Such a visual heuristic different from human visual perception merits further exploration, to gain better knowledge on how the model detect for COVID-19 and which features they consider most diagnostic. The discovery of these features would contribute to explaining the power of the model in COVID-19 testing, as well as assisting clinical doctors in discovering new visual indicators for COVID-19 infections for use in manual screening based on CT images.

## Discussion

Our study applied transfer learning on COVID-19 testing using CT images and discussed the impacts of various initialization parameters on the results, demonstrating that our model which were pre-trained on ImageNet21k has strong generalizability in terms of CT images. The proposed model provides an accuracy of 99.2% while detecting the COVID-19 cases. Compared to the neural architecture search model, our model shows the state-of-the-art performance, across all metrics we have described. These ensure that COVID-19-negative patients are correctly diagnosed as negative in the vast majority of cases, reduce probability of diagnosing COVID-19-negative cases as positive and reduce the burden on the health care system. Additionally, we examined the performance of the model with limited data and found that the model still perform satisfactorily. This shows that our model is still applicable with a limited data, which is characteristic of the real situation, where large and diverse datasets may not be readily available. Finally, we explored the relevant mechanism of COVID-19 testing using Grad-CAM visualization technique to make the proposed deep learning model more interpretable and explainable. The model performs performance validation through interpretability driven in a manner consistent with the radiologist’s interpretation for the CP. The investigation of normal and NCP CT images helps to explore new visual indicators to assist clinical doctors in further manual screening. The experiments demonstrate that our models are effective in COVID-19 testing. In future, we will pay attention to the evaluation of the severity of COVID-19 and attempt to discover more valuable information from CT images to combat the pandemic. We will further conduct explanatory analyses on the models, which will shed light on the detection mechanism of COVID-19, to identify key characteristics in the CT images and to facilitate the screening by clinical doctors. Although the system has good performance on public datasets, the work is still at theoretical research stage, and the models has not been validated in actual clinical routine. Therefore, we will test our system in the clinical routine and communicate with physicians to understand how they use it and their opinions about the models. Thus, we can further improve the models in our future work.

## Data Availability

The datasets analysed during the current study are available in the COVIDNet-CT repository, https://github.com/haydengunraj/COVIDNet-CT.
